# Body Sway Increases After Functional Inactivation of the Cerebellar Vermis by cTBS

**DOI:** 10.1007/s12311-015-0758-5

**Published:** 2016-01-16

**Authors:** Silvia Colnaghi, Jean-Louis Honeine, Stefania Sozzi, Marco Schieppati

**Affiliations:** 1 Department of Public Health, Experimental and Forensic Medicine, University of Pavia, Via Forlanini 2, 27100 Pavia, Italy; 2 Centro Studi Attività Motorie, Fondazione Salvatore Maugeri (IRCCS), Pavia, Italy

**Keywords:** Cerebellum, Stance, Sway, Magnetic stimulation, Sensorimotor integration, Visual shift

## Abstract

Balance stability correlates with cerebellar vermis volume. Furthermore, the cerebellum is involved in precise timing of motor processes by fine-tuning the sensorimotor integration. We tested the hypothesis that any cerebellar action in stance control and in timing of visuomotor integration for balance is impaired by continuous theta-burst stimulation (cTBS) of the vermis. Ten subjects stood quietly and underwent six sequences of 10-min acquisition of center of foot pressure (CoP) data after cTBS, sham stimulation, and no stimulation. Visual shifts from eyes closed (EC) to eyes open (EO) and vice versa were presented via electronic goggles. Mean anteroposterior and mediolateral CoP position and oscillation, and the time delay at which body sway changed after visual shift were calculated. CoP position under both EC and EO condition was not modified after cTBS. Sway path length was greater with EC than EO and increased in both visual conditions after cTBS. CoP oscillation was also larger with EC and increased under both visual conditions after cTBS. The delay at which body oscillation changed after visual shift was longer after EC to EO than EO to EC, but unaffected by cTBS. The time constant of decrease or increase of oscillation was longer in EC to EO shifts, but unaffected by cTBS. Functional inactivation of the cerebellar vermis is associated with increased sway. Despite this, cTBS does not detectably modify onset and time course of the sensorimotor integration process of adaptation to visual shifts. Cerebellar vermis normally controls oscillation, but not timing of adaptation to abrupt changes in stabilizing information.

## Introduction

Balance control under quiet stance depends upon continual integration of sensory inputs from visual, vestibular, and somatosensory receptors that help assess instantaneous orientation in space of the body [1, 2]. Unavailable information from any sense can result in instability due to mismatch of incoming sensory signals [3, 4]. The CNS is able to compensate to some extent by relying more upon the remaining information [5]. In this process of adjusting the sensory contributions to balance control, sensory reweighting occurs [6, 7].

The cerebellum is involved in precise control of motor processes at a high spatiotemporal resolution by fine-tuning sensorimotor integration [8–10] and by providing feedforward control mechanisms that are paramount in the control of stance [11, 12]. Animal studies on cerebellar functions for body balance stressed that the cerebellar vermis plays a role in integrating visual [13], proprioceptive [14], and cutaneous input from the paw [15–17]. Similarly, the human cerebellar vermis seems to co-operate in keeping the center of gravity within the limits required for stable upright standing [18]. It is also involved in processing self-motion perception [19, 20 for a review] and in the control of stance under critical conditions [21]. In keeping with these notions, standing balance deteriorates with age-related shrinkage of the vermis [22]; it is impaired in individuals with degenerative cerebellar disease, including spino-cerebellar ataxia [23, 24] and in chronic alcoholism, in which sway path length is selectively related to the volume of the vermis [25, 26].

Evidence for a cerebellar role in visual processing originates from anatomical and electrophysiological studies in monkeys and cats indicating the existence of cerebellar connections with the visual cortices. Visual inputs are conveyed to vermal lobules VI and VII and to the dorsal paraflocculus [27] and corticopontine projections derive from posterior parietal association cortices and from visual association cortices in the parastriate region [28, 29]. Measurement of human brain activity with resting-state functional MRI has allowed the connectivity between specific zones of the cerebellum and the rest of the brain including the visual cortices to be determined [30, 31]. Cerebellar patients show deficits in visual motion discrimination [32–37]. A recent study has demonstrated the causal role of the cerebellar vermis in visual motion processing in normal subjects [38].

Despite the central role of the cerebellum in controlling balance [39, 40] and its participation in sensory processing [41] having been widely discussed, cerebellar contribution to the processing of visual information for balance control in humans remains elusive. We aimed at investigating whether normal function of the cerebellar vermis contributes to the control of body sway and how it interacts with visual information availability. A corollary question was whether the time course of the changes in stabilometric variables in response to abrupt changes from no vision to vision or vice versa [42, 43] is under the control of cerebellar vermis. The rate dependency of most of the cerebellar activation sites in an imaging study by Claeys et al. [44] suggests that cerebellar participation is critical whenever a task has to be performed rapidly. This would fit with many clinical findings that have suggested a timing function for the cerebellum [45, 46]. Hence, we also posited that any role of the cerebellum both in minimizing sway and in speeding up the visuomotor integration process for balance control could be evidenced by inducing a transient functional impairment of the cerebellar vermis by transcranial magnetic stimulation (TMS). To this end, changes in stabilometric variables in response to abrupt changes in visual condition were recorded in normal participants standing with feet parallel on a soft pad after cerebellar continuous theta-burst stimulation (cTBS). The reason behind this procedure is that synaptic efficiency of the cerebral and cerebellar cortex is downregulated by cTBS [47–49].

## Materials and Methods

### Participants

Five females and five males (mean age 27.8 years ± 6.9 SD, height 172.4 cm ± 7.0 SD, weight 64.5 ± 8.7 kg) participated in the experiments. They were free from otological, neurological, or orthopedic abnormality, had normal or corrected visual acuity, and were naive to the experimental task. All procedures were carried out in accordance with Declaration of Helsinki with adequate understanding and written informed consent of the subjects. The research protocol had been approved by the local review board and ethical committee.

### Task and Procedure

Each subject underwent three recording sessions scheduled in random order and separated by 7 days: (i) no stimulation (nostim), (ii) cerebellar cTBS, and (iii) sham cTBS (sham). In each recording session, the experiments took place in a normally lit room. In cTBS and sham sessions, we started the recording soon after cerebellar or sham stimulation. Subjects stood quietly on a soft pad fixed over a force platform with the arms by their side, feet roughly parallel, and heels placed 5 cm apart. In this position, subjects performed a series of six trials of 10 min each. Subjects stood eyes open in front of the laboratory wall at a distance of 100 cm and were asked to focus on a target, a low contrast 5-cm wide emoticon, placed on the wall at eye level. They wore electronically controlled shutter goggles (Plato Visual Occlusion Spectacles, Translucent Technologies Inc., Toronto, Canada) that allowed sudden presentation or withdrawal of the visual scene. The goggles were controlled by the operator, by means of a TTL signal issued at unexpected delays from the onset of acquisition. The response time of the lenses to the voltage transient is approximately 1 ms to open and 3–5 ms to close (manufacturer’s specification). The TTL signal was stored in a PC in order to set the time of visual shift. In the open state (henceforth, eyes open (EO)), looking through the lenses was like looking through clear glass, while in the closed state (eyes closed (EC)), the lenses featured a translucent milky texture, which prevented the subject from perceiving visual information while the eyes remained illuminated and would not readapt to light when the lenses reopened. The goggles prevented peripheral vision because of the shape of their frame. The first change of the lenses state was presented after 30 s from the start of the acquisition. During each acquisition epoch a series of ten repetitions of the visual shift (in each direction) was presented: EC–EO shifts were alternated with EO–EC shifts in sequence at pseudo-random delays varying from 30 to 34 s. The overall duration of the experimental session, including preparation of the subject for recording, varied from 60 to 75 min. The number of repetitions allowed offline averaging of a sufficient number of force-platform traces (normally about 60) to obtain an average trace on which to reliably estimate the level of sway and the time delay, following the sensory shift, at which modifications occurred in stance variables. All offline analyses were made with a time window of 30 s for each trial, where the visual shift was set at midpoint.

### Cerebellar Magnetic Stimulation

A magnetic stimulator (Super Rapid^2^, MagStim Company, Whitland, UK), connected with a figure-of-eight coil with a diameter of 70 mm, was used to deliver cTBS over the scalp site corresponding to the cerebellar vermis. TMS was applied over the midline cerebellum using the same scalp coordinates (1 cm inferior to the inion) adopted in previous studies. In these studies, MRI reconstruction and neuronavigation systems showed that cerebellar TMS in such a location predominantly targets the posterior and superior lobules of the vermis [50]. The coil was positioned tangentially to the scalp, with the handle pointing superiorly. The exact coil position was marked by an inking pen to ensure an accurate positioning of the coil throughout the experiment. The stimulating coil was held by hand, and coil position was continuously monitored throughout the experiment. The magnetic stimulus had a biphasic waveform with a pulse width of about 300 μs. During the first phase of the stimulus, the current in the center of the coil flowed toward the handle. Three-pulse bursts at 50 Hz repeated every 200 ms for 40 s, equivalent to cTBS in Huang et al. [47], were delivered over midline cerebellum (600 pulses) at 100 % of resting motor threshold (RMT), defined as the lowest intensity that produced MEPs of >50 μV in at least five out of ten trials in a relaxed muscle [51]. RMT was assessed by stimulating over the motor cortex of the left hemisphere, and the electromyogram was recorded from the FDI muscle of the right hand using pre-gelled, self-adhesive electrodes (Ambu Neuroline 720). The active electrode was placed over the muscle belly and the reference electrode over the metacarpophalangeal joint of the index finger. Responses were amplified and recorded using the same magnetic stimulator through filters set at 20 and 10 kHz. In our subjects, stimulation intensity for cTBS proved to be 53.5 ± 5.4 % (mean ± SD) of the maximum stimulator output. Sham stimulation was delivered through the same focal coil angled at 90° with only the edge of the coil resting on the scalp. Stimulus intensity was set at only 40 % RMT for the first dorsal interosseous (FDI). The order of presentation (cTBS or sham) was counterbalanced across subjects. This stimulation intensity, while ineffective in inducing any neural activation or unpleasant sensations [52–54], ensured adequate noise. The tactile scalp sensation induced by the tilted arrangement of the coil was indistinguishable from that of the real cTBS.

### Detection and Analysis of the CoP by Stabilometry

**Fig. 1:**
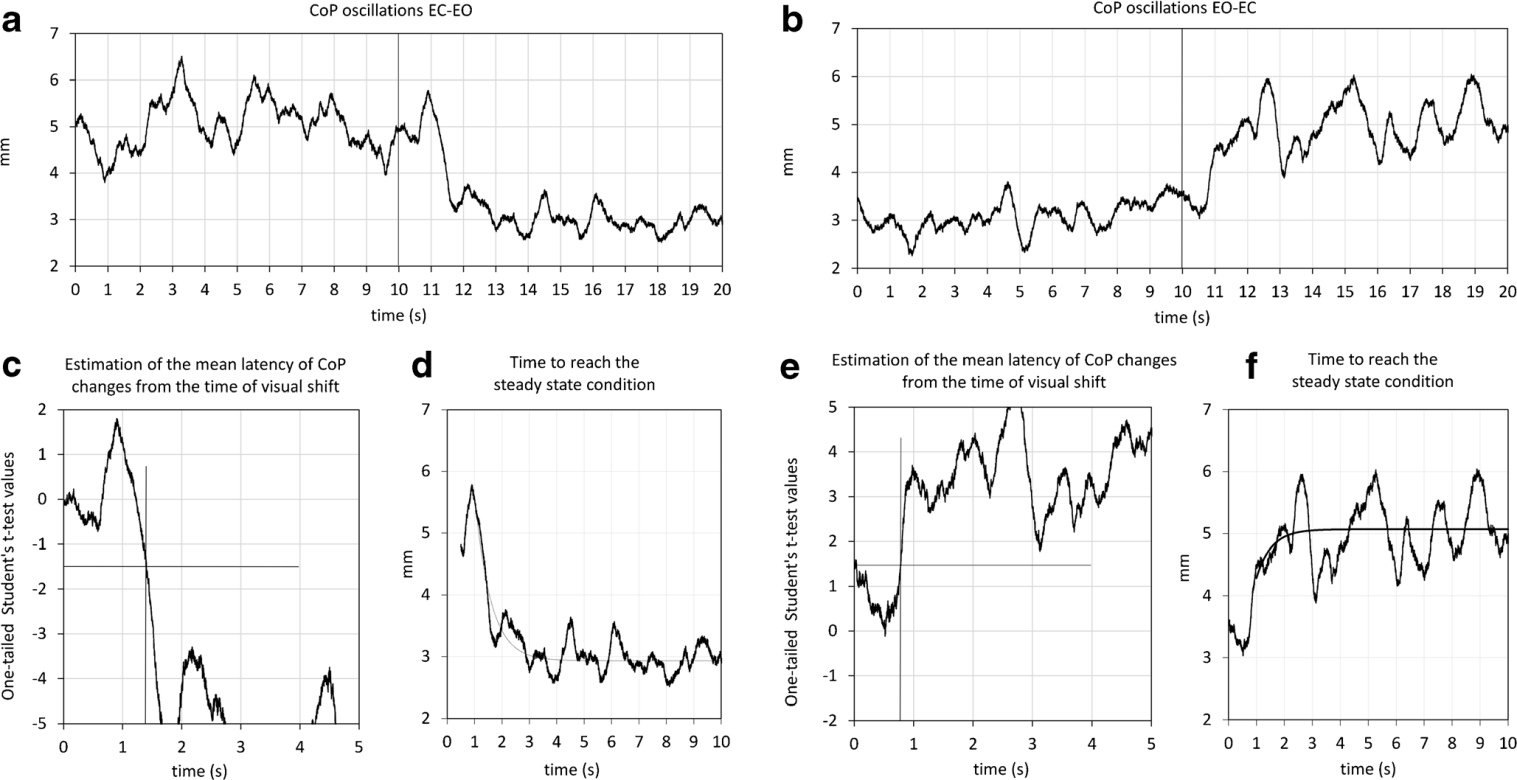
Average time profile of the center of pressure (CoP) oscillation around the time of visual shift. **a**, **b** The mean A-P CoP oscillation of one subject during the EO–EC and EC–EO trials. Time flows from left to right starting with EO (**a**) and with EC (**b**), until *t* = 10 s, at which visual condition changes. **c**, **e** show the probability of rejecting the zero hypothesis regarding the mean difference between the two oscillation traces after the visual condition change. The EO trace (**c**) becomes significantly different from the EC trace when the probability drops below the line at *y* = 1.67; vice versa for **e**. The corresponding intervals estimate the latency of the stabilizing effect of addition (**a**, **c**) or withdrawal of vision (**b**, **e**). The CoP traces are fitted with an exponential curve, the time constant of which is calculated for estimating the time to recovery steady state

The ground reaction force was acquired by a force platform (Kistler K9286BA, Switzerland) at 560 Hz and stored on a PC for offline analysis (SMART-D, BTS, Italy). The platform output was the instantaneous position of the center of pressure (CoP) along the sagittal and frontal axis in the horizontal plane during the standing trials (with or without vision). The CoP position was evaluated before rectification. To quantify the sway amplitude on both sagittal (anteroposterior, A-P) and frontal (mediolateral, M-L) axes, the CoP position trace was high-pass filtered and rectified (bidirectional third-order Butterworth) with a cutoff frequency of 0.1 Hz for subsequent averaging (Fig. 1a, b). Typically, very slow frequencies are connected with small and slow forward body displacement to a more secure position that is sometimes observed on closing the eyes, and with recovery to the original stance orientation eyes open [55, 56]. The high-pass filter was chosen to eliminate these slow frequencies from the CoP and highlight the relatively faster CoP oscillations. Henceforth, we refer to the filtered and rectified CoP trace as amplitude of body oscillation. For every trial of every subject, the mean A-P and M-L CoP position and oscillation under EC and EO condition at steady state were calculated by averaging each trace during the last 10 s of the EC–EO and EO–EC condition (steady state prior to visual shift).

### Estimation of the Mean Latency of the CoP Changes from the Time of Visual Shift

For each subject and direction of visual shift, the latency following the sensory shift, at which body sway diminished or increased depending on the visual shift direction, was estimated on the averaged traces of the 60 trials containing the visual shift (EC–EO or EO–EC). Changes in CoP (position and oscillation) pattern after the time of changing visual condition were estimated by comparing the average traces for each condition around the time of visual shift using the one-tailed Student’s *t* test according to a published procedure used in this laboratory [43]. Briefly, we divided the trial at the time of visual condition change and calculated the mean value of the average trace in the 10 s preceding the change. We then used a paired *t* test for comparison between this constant value and the individual values following the visual shift, with the number of samples increasing progressively from the first point after the shift until the significance level was reached. The latency of CoP changes was thus the duration of the interval elapsing from the visual condition change to the time at which the *t* value of the above comparisons bypassed the critical value of *t* = 1.67, corresponding to a 0.05 probability of rejecting the zero hypothesis, and remained above it for at least 100 ms (Fig. 1c, e).

### Time to Reach Steady-State Condition

Following the change in CoP occurring immediately after the sensory shift, the signals gradually reached the steady state pertaining to the new sensory condition. We hypothesized that the trace could be fitted with an exponential model because it roughly displayed an initially rapid variation and tended to plateau over time. The mean traces of A-P and M-L CoP sway of each subject were fitted with an exponential model (*y* = *A* + *Be* ^–*t*/*τ*^) by the Excel^®^ Solver utility. Tau (*τ*) is the time constant of the recovery, *A* is the value at steady state, *A* + *B* is the intercept with the ordinate. *A*, *B*, and *τ* were computed by using the minimum sum squared algorithm by the iterative conjugate gradient method of the utility. The curves were fitted from *t* = onset latency of CoP changes after visual shift until the end of a 10-s time window (Fig. 1d, f).

### Comparison Between Cerebellar Stimulation, Sham, and No Stimulation Trials

For each trial and subject, the mean A-P and M-L position were computed for both EC and EO conditions, at steady state. In order to account for the possible recovery of the cerebellar function toward the end of the recording session (about 1 h), we considered separately the first and the last 30 trials in each session (first and last recording epoch). The CoP position mean values on the sagittal axes (A-P) were analyzed by a 3 × 2 × 2 repeated measures ANOVA (experimental session: [nostim, cTBS, sham] × visual condition [EC, EO] × recording epoch [first, last]). The mean amplitude of A-P and M-L CoP oscillations were computed for both EC and EO conditions, at steady state. We considered separately the first and the last recording epoch and analyzed the data by a 3 × 2 × 2 × 2 repeated measures ANOVA (experimental condition: [nostim, cTBS, sham] × visual condition [EC, EO] × recording epoch [first–last] × CoP axis [A-P, M-L]). The mean latencies of the change in A-P and M-L CoP oscillations in response to the visual shift were analyzed by a 3 × 2 × 2 repeated measure ANOVA (experimental condition: [nostim, cTBS, sham] × direction of visual shift [EC–EO, EO–EC] × CoP axis [A-P, M-L]). The time constants were analyzed by a 3 × 2 × 2 repeated measure ANOVA (experimental condition: [nostim, cTBS, sham] × direction of visual shift [EC–EO, EO–EC] × CoP axis [A-P, M-L]). Mean latencies and time constants variables were calculated and analyzed from the whole of 60 trials of each recording session, in order to have enough repetitions for an average trace on which to reliably estimate the level of sway and the time delay, following the sensory shift, at which modifications occurred in stance variables. All post hoc tests were made using the Fisher’s LSD test. The software package Statistica (StatSoft, USA) was used for all statistical analyses.

## Results

### Steady-State Variables

#### Sway Path Length

**Fig. 2:**
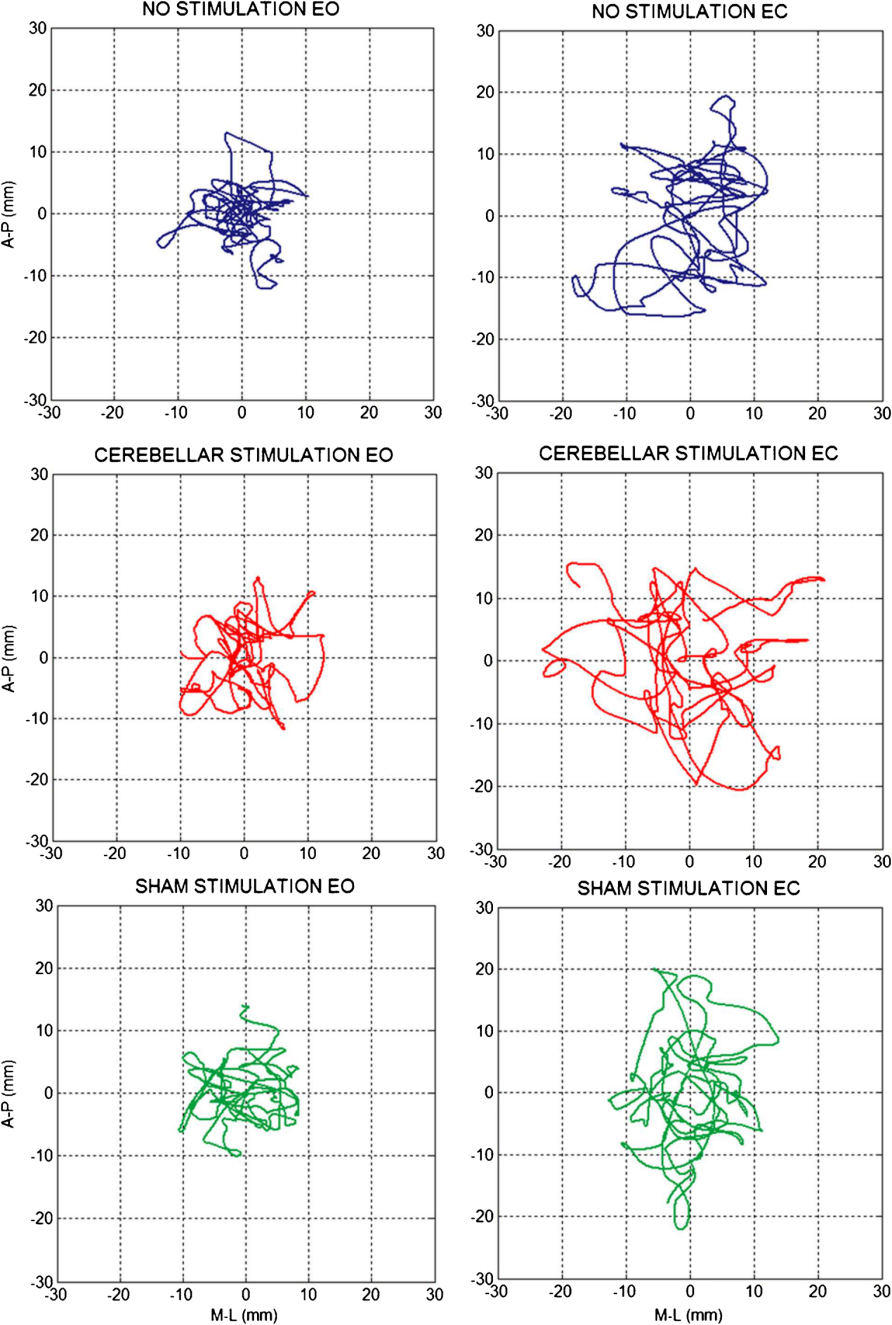
CoP sway of a representative subject. Center of foot pressure displacement on the horizontal plane recorded during a 15-s period of quiet stance (steady state), feet parallel, under EO (*left panels*) and EC (*right panels*) conditions in each experimental session: no stimulation (*first row*), cerebellar cTBS stimulation (*second row*), and sham stimulation (*third row*). CoP sway has larger amplitude during the EC than EO period and increases in the recording made after cerebellar stimulation

**Fig. 3:**
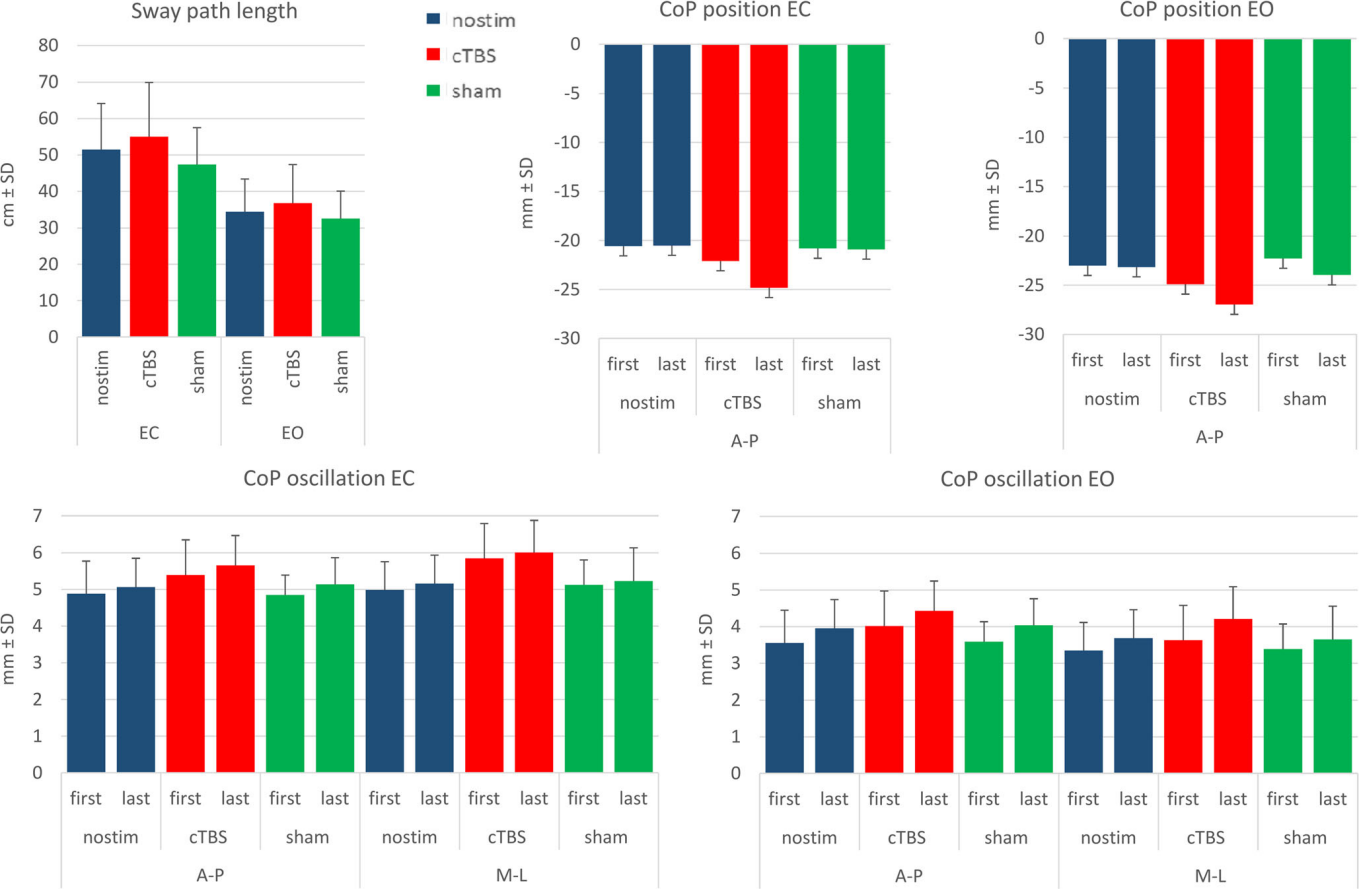
CoP position and oscillation. Sway path mean length, CoP mean position and mean oscillation amplitude in the three experimental sessions. The values were obtained by averaging the mean values of all subjects in the first and last 30 trials of each recording session, EO and EC. Sway path length and A-P and M-L CoP oscillation are significantly larger under EC than EO conditions. Within each condition, they are larger after cerebellar stimulation. CoP A-P position showed a shift forward with EC (zero corresponds to the middle of the platform, positive values correspond to forward position in the sagittal plane)

The mean values of sway path length (Figs. 2 and 3, Table 1) were significantly greater with EC than EO (visual condition effect, *p* < 0.001). The mean values of sway path length (both EC and EO) also increased significantly after cerebellar stimulation with respect to sham stimulation and no stimulation conditions (experimental condition effect, *p* = 0.003); there was no statistical interaction (experimental condition × visual condition interaction, *p* = 0.19). Post hoc test showed a significant difference in sway path length after cerebellar stimulation with respect to the other conditions (cTBS vs nostim, *p* = 0.02; cTBS vs sham, *p* < 0.001), while no difference was found between no stimulation and sham stimulation condition (*p* = 0.13).

**Table 1:** Sway path length (3 experimental condition × 2 visual condition ANOVA)

Effects	*F*	*p* value	Fisher’s test	
			Comparisons	*p* value
Experimental condition	8.126_(2,18)_	0.003	cTBS > nostim	0.027
			cTBS > sham	<0.001
			nostim = sham	0.127
Visual condition	85.556_(1,9)_	<0.001		
Experimental condition × visual condition	1.830_(2,18)_	0.189		

Experimental condition: no stimulation (nostim), cerebellar stimulation (cTBS), sham stimulation (sham); visual condition: eyes closed (EC), eyes open (EO)

#### CoP Position

The average CoP position (Fig. 3, Table 2) shifted slightly forward in the transition from EO to EC and vice versa (visual condition effect, *p* = 0.043), both in the first and last recording epoch (recording epoch effect, *p* = 0.222). The mean values of CoP position, EC and EO, were not significantly modified after cerebellar stimulation (experimental condition effect, *p* = 0.292, experimental condition × visual condition interaction, *p* = 0.951), neither in the first or in the second recording epoch (experimental condition × recording epoch interaction, *p* = 0.243).

**Table 2:** CoP position on the sagittal axis (3 experimental condition × 2 visual condition × 2 recording epoch ANOVA)

Effects	*F*	*p* value
Experimental condition	1.317_(2,18)_	0.292
Visual condition	5.510_(1,9)_	0.043
Recording epoch	1.633_(1,9)_	0.233
Experimental condition × visual condition	0.051_(2,18)_	0.951
Experimental condition × recording epoch	1.533_(2,18)_	0.243
Visual condition × recording epoch	0.445_(1,9)_	0.521
Experimental condition × visual condition × recording epoch	1.563_(2,18)_	0.236

Experimental condition: no stimulation (nostim), cerebellar stimulation (cTBS), sham stimulation (sham); visual condition: eyes closed (EC), eyes open (EO); recording epoch: first 30 trials (first), last 30 trials (last)

#### CoP Oscillation

**Fig. 4:**
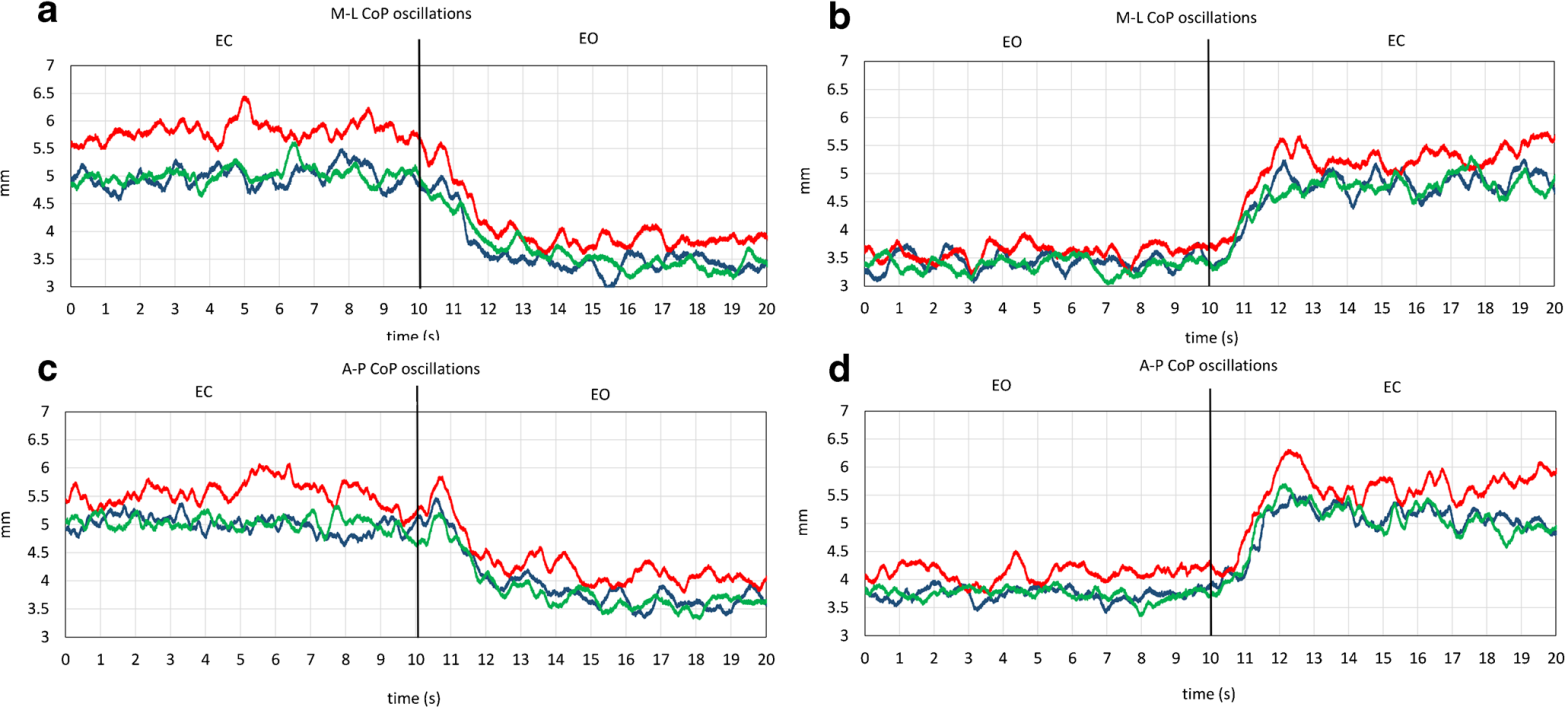
CoP oscillation traces. Grand means of all subjects of M-L and A-P CoP oscillation during the EO–EC and EC–EO trials, after no stimulation (*blue line*), after cerebellar stimulation (*red line*), and after sham stimulation (*green line*). In **a**, **c**, CoP oscillation is small with EO and increases after visual occlusion; in **b**, **d**, the oscillation decreases as soon as vision is allowed. Note that, in all four panels, the red traces (cTBS trials) are positioned above the *blue* (no stimulation trials) and the *green* (sham stimulation trials) traces (color figure online)

**Fig. 5:**
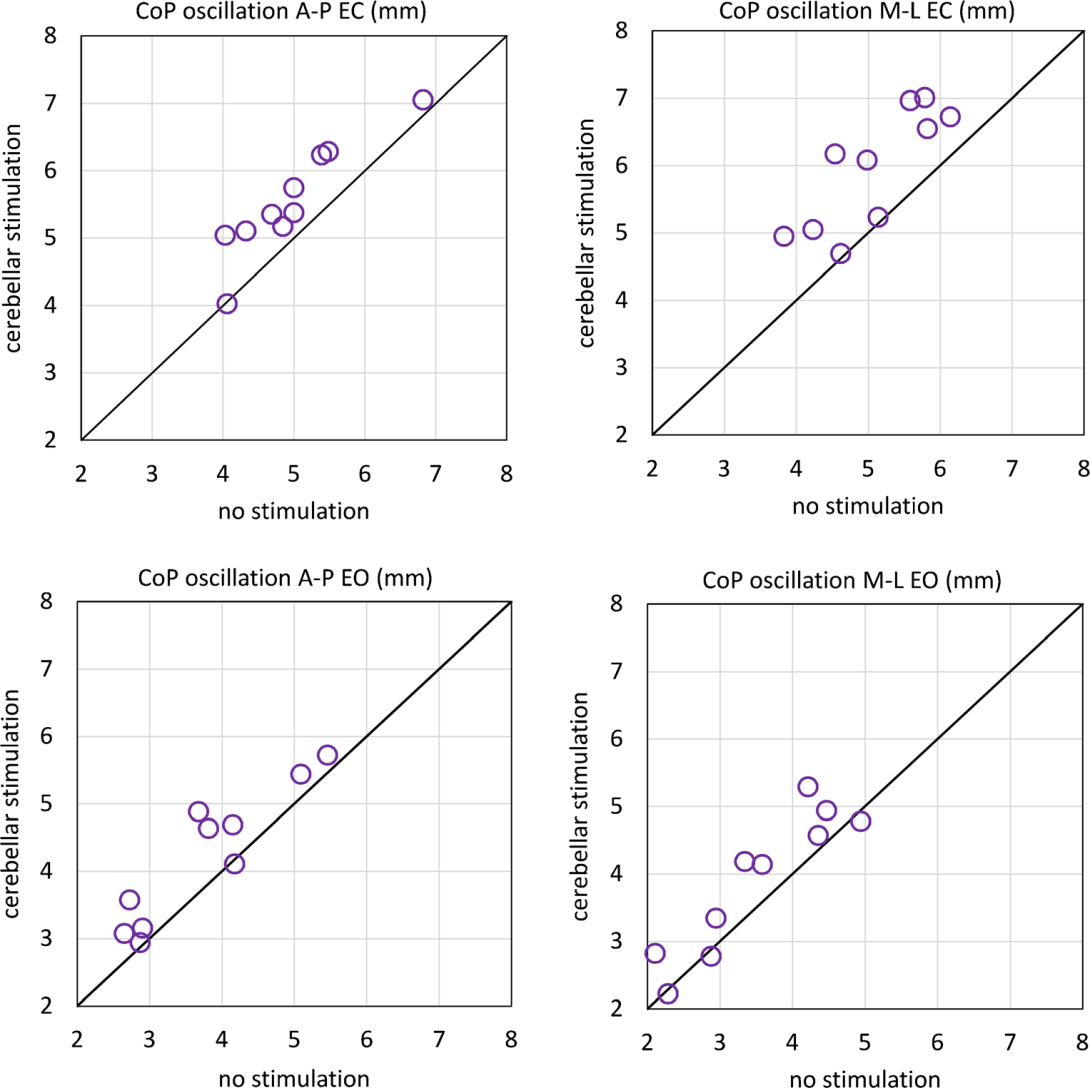
Comparisons of no stimulation and cerebellar stimulation on the mean CoP oscillation values in each subject. CoP mean oscillation amplitude during quiet stance, feet parallel. The values were obtained by averaging the mean values of each subjects, collected during a 10-s steady-state period under EC (*top panels*) and EO conditions. In most subjects, CoP oscillation amplitude is larger after cerebellar stimulation than no stimulation, under both EO and EC conditions

The mean values of CoP oscillation (Figs. 3 and 4, Table 3) were significantly greater with EC (visual condition effect, *p* < 0.001), particularly in the frontal plane (CoP axis effect, *p* = 0.89; visual condition × CoP axis interaction, *p* < 0.001). CoP oscillation mean values increased in the second half of the session (recording epoch effect, *p* = 0.012), similarly in both directions (CoP axis × recording epoch interaction, *p* = 0.554) and in each experimental condition (experimental condition × recording epoch interaction, *p* = 0.771), particularly in the EO condition (visual condition × session time interaction, *p* = 0.016). The mean values of both EC and EO CoP oscillation (Fig. 5) increased significantly after cerebellar stimulation (experimental condition effect, *p* < 0.001, experimental condition × visual condition interaction, *p* = 0.08), and this effect was significant both in the sagittal and in the frontal plane (experimental condition × CoP axis interaction, *p* = 0.46). Post hoc test showed a difference between the CoP oscillations after cerebellar stimulation and the other conditions (cTBS versus nostim, *p* < 0.001; cTBS vs sham, *p* < 0.001), while no difference was found between no stimulation and sham condition (*p* = 0.712). Since, on the basis of the mean values, cerebellar stimulation effects in the frontal plane seemed larger with EC, we verified this point by performing a 2 (visual condition) × 2 (CoP axis) ANOVA (Table 4) on the difference between cerebellar stimulation and control (means of no stimulation and sham) values (visual condition effect *p* = 0.033; visual condition × CoP axis interaction, *p* = 0.033). Post hoc test proved that cerebellar stimulation effect was significantly greater in the frontal plane with EC (M-L EC vs M-L EO, *p* = 0.001; M-L EC vs A-P EC, *p* = 0.064).

**Table 3:** CoP oscillation (3 experimental condition × 2 visual condition × 2 recording epoch × 2 CoP axis ANOVA)

Effects	*F*	*p* value	Fisher’s test	
			Comparisons	*p* value
Experimental condition	12.740_(2,18)_	<0.001	cTBS > nostim	<0.001
			cTBS > sham	<0.001
			nostim vs sham	0.712
Visual condition	133.508_(1,9)_	<0.001		
Recording epoch	9.865_(1,9)_	0.012		
CoP axis	0.019_(1,9)_	0.892		
Experimental condition × visual condition	2.959_(2,18)_	0.077		
Experimental condition × recording epoch	0.263_(2,18)_	0.771		
Visual condition × recording epoch	8.698_(1,9)_	0.016	EC last > EC first	0.004
			EO last > EO first	<0.001
			EC first > EO first	<0.001
			EC first > EO last	<0.001
			EC last > EO first	<0.001
			EC last > EO last	<0.001
Experimental condition × CoP axis	0.813_(2,18)_	0.459		
Visual condition × CoP axis	47.566_(1,9)_	<0.001	EC M-L > EC A-P	0.002
			EO A-P > EO M-L	<0.001
			EC A-P > EO A-P	<0.001
			EC A-P > EO M-L	<0.001
			EC M-L > EO A-P	<0.001
			EC M-L > EO M-L	<0.001
Recording epoch × CoP axis	0.377_(1,9)_	0.554		
Experimental condition × visual condition × recording epoch	0.374_(2,18)_	0.693		
Experimental condition × visual condition × CoP axis	3.228_(2,18)_	0.063		
Experimental condition × recording epoch × CoP axis	1.372_(2,18)_	0.279		
Experimental condition × recording epoch × CoP axis	0.726_(1,9)_	0.416		
Experimental condition × visual condition × recording epoch × CoP axis	1.012_(2,18)_	0.383		

Experimental condition: no stimulation (nostim), cerebellar stimulation (cTBS), sham stimulation (sham); visual condition: eyes closed (EC), eyes open (EO); CoP axis: medio-lateral (M-L), antero-posterior (A-P); recording epoch: first 30 trials (first), last 30 trials (last)

**Table 4:** CoP oscillation difference between cerebellar stimulation and control (2 visual condition × 2 CoP axis ANOVA)

Effects	*F*	*p* value	Fisher’s test	
			Comparisons	*p* value
CoP axis	0.262_(1,9)_	0.621		
Visual condition	6.364_(1,9)_	0.033		
CoP axis × visual condition	6.322_(1,9)_	0.033	M-L EC > M-L EO	0.001
			M-L EC > A-P EC	0.064
			M-L EC > A-P EO	0.014
			A-P EC > A-P EO	0.369
			A-P EC > M-L EO	0.040
			M-L EO > A-P EO	0.181

Visual condition: eyes closed (EC), eyes open (EO); CoP axis: medio-lateral (M-L), antero-posterior (A-P)

### Integration Latency and Reweighting Time

#### Mean Latency of CoP Changes from the Time of Visual Shift

**Fig. 6:**
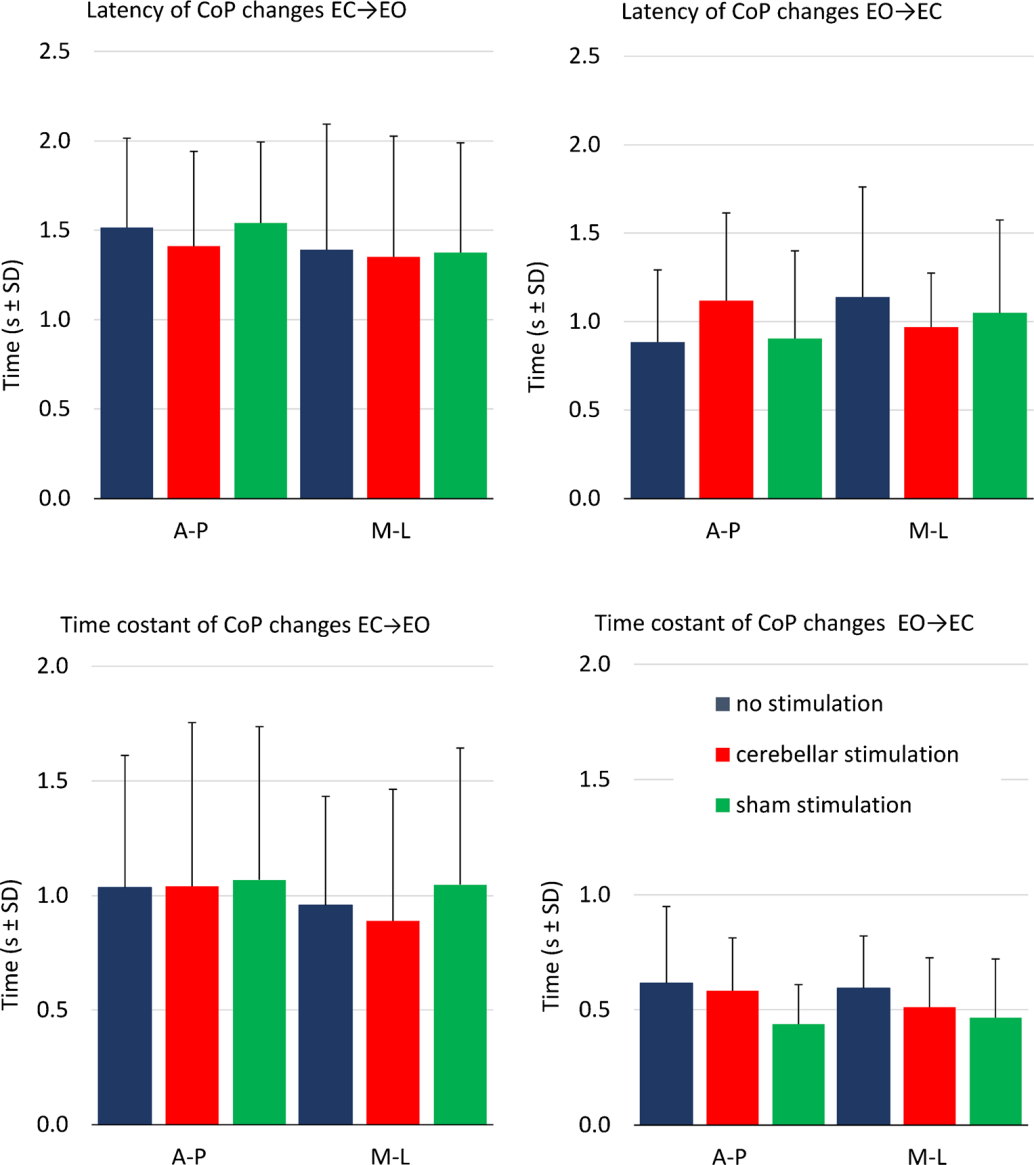
Mean latency and time constant of CoP changes after the visual shift. Mean values of the time-to-recovery to the steady-state value and of the time constant of CoP A-P and M-L oscillation, when vision is occluded (EO–EC) or allowed (EC–EO). Both time variables were significantly longer when vision was added (EC to EO shifts, *left panels*) than withdrawn (EO to EC shift, *right panels*) and were not affected by cerebellar stimulation

The delay of the onset of oscillation changes after the visual shift (Fig. 6, Table 5) was significantly longer when vision was added (EC to EO) than withdrawn (EO to EC shift) (visual shift effect, *p* = 0.004) but was not affected by cerebellar stimulation (experimental condition × visual shift interaction, *p* = 0.49; experimental condition × CoP axis interaction, *p* = 0.35).

**Table 5:** Latency of CoP changes (3 experimental condition × 2 visual shift × 2 CoP axis ANOVA)

Effects	*F*	*p* value
Experimental condition	0.104_(2,18)_	0.901
CoP axis	0.112_(1,9)_	0.746
Visual shift	14.064_(1,9)_	0.004
Experimental condition × CoP axis	1.099_(2,18)_	0.355
Experimental condition × visual shift	0.748_(2,18)_	0.487
CoP axis × visual shift	2.386_(1,9)_	0.157
Experimental condition × CoP axis × visual shift	0.550_(2,18)_	0.586

Experimental condition: no stimulation (nostim), cerebellar stimulation (cTBS), sham stimulation (sham); CoP axis: medio-lateral (M-L), antero-posterior (A-P); visual shift: EC to EO, EO to EC

#### Time Constant of CoP Changes from the Time of Visual Shift

The time constant of the oscillation changes after the visual shift (Fig. 6, Table 6) was significantly different between the EC to EO and the EO to EC conditions (visual shift effect, *p* = 0.006) and was not affected by cerebellar stimulation (experimental condition effect, *p* = 0.9; experimental condition × visual shift interaction, *p* = 0.45; experimental condition × CoP axis interaction, *p* = 0.72).

**Table 6:** Time constant of CoP changes (3 experimental condition × 2 visual shift × 2 CoP axis ANOVA)

Effects	*F*	*p* value
Experimental condition	0.103_(2,18)_	0.902
CoP axis	0.436_(1,9)_	0.526
Visual shift	12.751_(1,9)_	0.006
Experimental condition × CoP axis	0.336_(2,18)_	0.720
Experimental condition × visual shift	0.828_(2,18)_	0.453
CoP axis × visual shift	0.217_(1,9)_	0.653
Experimental condition × CoP axis × visual shift	0.009_(2,18)_	0.991

Experimental condition: no stimulation (nostim), cerebellar stimulation (cTBS), sham stimulation (sham); CoP axis: medio-lateral (M-L), antero-posterior (A-P); visual shift: EC to EO, EO to EC

## Discussion

Theta-burst stimulation (TBS) uses three pulses of stimulation given at 50 Hz, repeated every 200 ms. A 2 s train of TBS is repeated every 10 s for a total of 190 s (600 pulses) in the intermittent TBS paradigm (iTBS), while a 40 s train of uninterrupted TBS is given (600 pulses) in continuous TBS paradigm (cTBS). These TMS protocols may be used for cerebellar stimulation to modulate the excitability of the underlying cerebello-thalamo-cortical pathways that are linked with distinct intracortical M1 circuits [57, 58]. The pattern of delivery of TBS is crucial in determining the direction of change in synaptic efficiency [48]: a facilitation of MEP size follows iTBS and lasts for about 15 min, whereas a reduction of MEP size follows cTBS and lasts for nearly 60 min. Following 40 s of cTBS (bursts containing three pulses at 50 Hz repeated continuously at 200-ms intervals), the nervous circuits target of the cerebellar activity undergo a modulation lasting up to 1 h [47]. The cerebellar function in our experiments did not recover toward the end of the session. CoP oscillations increased in the second half of the trials in each experimental condition, and we attributed this effect to the development of subjects’ exhaustion.

Distinctive clinical signs of cerebellar damage are balance abnormalities characterized by increased postural sway, excessive or inappropriate responses to perturbations, poor control of equilibrium during motions of other body parts, and abnormal oscillations of the trunk [59–62]. Balance deficits depend on lesion location [60, 63]. For instance, vermal and paravermal cortical areas are concerned primarily with mechanisms that modify extensor muscle tone for postural control [15, 64–66]. Vestibular nuclei also project to the posterior vermis and contribute to postural equilibrium [8, 67, 68].

Vision is the most important sensory input for detecting sway in unchallenged conditions [69], and its effect becomes relevant under more critical conditions like standing on a compliant surface that challenges stability, as in our case [70]. Standing on foam increases anteroposterior torque variance with EC by more than 100 % and produces a multidirectional postural instability [71].

In our experiments, the effect on sway of presence or absence of vision was obvious. We therefore measured body oscillation EO and EC before and after cTBS cerebellar stimulation in subjects standing on foam. cTBS over the cerebellar vermis produced increased sway in the sagittal and in the frontal plane under both visual conditions. We also evaluated the role of the cerebellum in the timing of the sensory reweighting process accompanying and following addition or withdrawal of vision. On allowing or occluding vision, decrements and increments in CoP oscillation occurred within about 2 s, and the time course of recovery to steady state was about 1 s. These results are in keeping with findings described in previous papers [42, 43]. However, despite resulting in increased sway attributable to disruption of the cerebellar function, cTBS over the cerebellar vermis did not significantly modify the delay of the onset and the time course of the sensory motor integration and of the reweighting processes involved in adaptation of stance control after sudden addition of vision.

### Increased Sway by cTBS

The cerebellum adjusts the input–output coupling of postural and oculomotor reflexes according to somatosensory and vestibular regulatory signals, in order to stabilize posture [72]. Such sensorimotor coupling requires an extraordinary computational effort, due to the large number of reflex responses and voluntary activations possibly generated in response to a given sensory input or motor command. It is conceivable that, without the cerebellum, the remaining parts of the motor systems are still able to perform some form of sensorimotor tuning. However, this would be less efficient, due to the loss of computational power [8]. Our findings are in keeping with this theory.

Sway path increased after cerebellar cTBS by about 7 %, under both EC and EO conditions. CoP oscillation increased of about 10 % EC and 9 % EO (A-P and M-L axes collapsed). Oscillation was therefore the most sensitive parameter for detecting increased body sway after cTBS. This is in keeping with the data collected after alcohol acute intoxication [73]. Remarkably, in our subjects, the Romberg Quotient (EC/EO) calculated for the oscillation parameter proved to be around 1.4 for both A-P and M-L oscillation, under both control condition and after cTBS.

This result of increased sway in the sagittal and frontal plane as a consequence of cTBS over the cerebellar vermis is in keeping with the induction of a transient perturbation of the cerebellar function. This directly shows that (i) the cerebellar vermis plays a pivotal role in the standing body’s stabilization. Moreover, since increased sway on cTBS was detectable both EC and EO, (ii) cerebellar processing of proprioceptive and vestibular inflow is deteriorated by cTBS. This effect is largely independent from vision. In fact, the effect of cerebellar cTBS in our experiment was larger EC than EO but was definitively present in both visual conditions. A comparison of the sway pattern of subjects after acute ethanol ingestion [74] with that of our subjects after cTBS suggests that the acute effect of alcohol resembles that of cTBS. It seems therefore that cTBS produces a functional impairment of the spinocerebellum.

### No Significant Effect of cTBS on the Time to Incorporate Visual Changes

Many fundamental and clinical findings have suggested a timing function for the cerebellum [50]. Hence, we specifically aimed at evaluating the role of the cerebellum in the timing of the integration and sensory reweighting process accompanying and following the shift in vision. In our hands, the hypothesis that inactivation of the cerebellar midline would have degraded the timing of the integration of stabilizing visual information was not verified. On allowing or occluding vision, decrements and increments in CoP oscillation occurred within about 1–2 s, and the time course of recovery to steady state was about 1 s, regardless of cTBS. These periods proved to be compatible with those already published in normal subjects standing in tandem position under a similar condition of sensory addition or withdrawal [42, 43, 74]. Both the latency to the earliest changes in oscillation and the period to recover to the appropriate steady state were not affected by cTBS on either addition or withdrawal of vision.

Therefore, in our experiments, magnetic stimulation likely targeted the cerebellar regions mainly involved in postural adjustment and not the ones responsible for processing the visual stimulus. In this line, the relatively long latency from the visual shift to the earliest changes in sway suggests the possibility that the process of visual integration for balance stabilization occurs at cortical level, since the cortex is certainly involved in controlling critical postures [75–82].

In a study using PET to investigate brain activation during maintenance of standing posture, shifting from EO to EC resulted in significant activation in the bilateral middle frontal gyri without significant cerebellar activation [21]. Interestingly, in patients with spinocerebellar ataxia type 6, in spite of clear-cut increase in body sway, habituation properties to new visual, vestibular, and proprioceptive inputs were not different from controls for all three sensory modalities [24], again indicating that temporal aspects of sensory integration may not be accomplished by the midline cerebellum. Imaging studies in humans and single cell recordings in primates underscore the prominent role of the premotor and parietal cortices [83–85] and the striato-pallido-thalamo-cortical circuit [86] as sensorimotor interface, supporting the hypothesis that these structures might be involved in the mechanisms of shifting the reference frame on the basis of the available sensory information.

The visual cortex itself may play a role in the integration of the visual inflow for balance control. Stimulation of the medial bank of the suprasylvian area elicits both mossy and climbing fiber responses, mainly in the vermis: direct cortico-pontine and indirect long-latency cortico-olivary projections are suggested to convey the respective responses [87, 88]. In turn, the inferior olivary nucleus has a substantial projection to the vermis and many axons branch to both the anterior and posterior part of the vermis, as a likely substrate of different segments’ coordination [89].

The most parsimonious explanation would be that the cerebellar vermis efferent system is involved in the active maintenance of body balance based on the continuous inflow from different inputs systems from the spinal cord, likely relayed onto the lateral reticular nucleus which provides the major mossy fiber input to cerebellum from spinal interneuronal systems [90], while its reweighting function would be under cortical control and wait for the trigger to come from the cortical association areas.

## Conclusions

cTBS over the cerebellar vermis produced increased sway in the sagittal and frontal plane. This shows that the cerebellar vermis plays a pivotal role in the standing body’s stabilization. Further, since increased sway on cTBS was detectable both with EC and with EO, cerebellar processing of proprioceptive and vestibular inflow is deteriorated by cTBS. Therefore, availability of vision is not enough to counteract the perturbed cerebellar control over body oscillation, even if the stabilizing effect of vision may be relayed by the cerebellar vermis.

Despite resulting in an increased sway, cTBS over the cerebellar vermis did not modify either latency of onset or time course of the sensory motor integration processes involved in adaptation of stance after sudden addition or withdrawal of vision. Hence, neural structures other than the cerebellum may be the site of the first step in visual information processing. These structures may be normally responsible for the delay in the onset of sway changes on addition or removal of vision, and send to the cerebellum the signals that induce reweighting of the vestibular and proprioceptive loops travelling through the vermis. Therefore, the midline cerebellum may not accomplish the temporal aspects of integration of a new sensory state. The inferior olivary nucleus may be the relay of the descending cortical information signaling sensory shift.
